# Multi-omic profiles of *Sorghum* genotypes with contrasting heat tolerance connect pathways related to thermotolerance

**DOI:** 10.1093/jxb/erae506

**Published:** 2024-12-19

**Authors:** Alexander Watson-Lazowski, Francisco Javier Cano, Mikael Kim, Urs Benning, Fiona Koller, Barbara George-Jaeggli, Alan Cruickshank, Emma Mace, David Jordan, Mathieu Pernice, Charles Warren, Oula Ghannoum

**Affiliations:** ARC Centre of Excellence for Translational Photosynthesis, Hawkesbury Institute for the Environment, Western Sydney University, 2753, Sydney, NSW, Australia; Harper Adams University, Newport, TF10 7AA, UK; ARC Centre of Excellence for Translational Photosynthesis, Hawkesbury Institute for the Environment, Western Sydney University, 2753, Sydney, NSW, Australia; Instituto de Ciencias Forestales (ICIFOR-INIA), CSIC, Carretera de la Coruña km 7.5, 28040, Madrid, Spain; University of Technology Sydney, Climate Change Cluster, 2007, Ultimo, NSW, Australia; ARC Centre of Excellence for Translational Photosynthesis, Hawkesbury Institute for the Environment, Western Sydney University, 2753, Sydney, NSW, Australia; ARC Centre of Excellence for Translational Photosynthesis, Hawkesbury Institute for the Environment, Western Sydney University, 2753, Sydney, NSW, Australia; ARC Centre of Excellence for Translational Photosynthesis, Queensland Alliance for Agriculture and Food Innovation, University of Queensland, 4067, Brisbane, QLD, Australia; Department of Agriculture and Fisheries (DAF), Hermitage Research Facility, Agri-Science Queensland, 4370, Warwick, QLD, Australia; ARC Centre of Excellence for Translational Photosynthesis, Queensland Alliance for Agriculture and Food Innovation, University of Queensland, 4067, Brisbane, QLD, Australia; ARC Centre of Excellence for Translational Photosynthesis, Queensland Alliance for Agriculture and Food Innovation, University of Queensland, 4067, Brisbane, QLD, Australia; University of Technology Sydney, Climate Change Cluster, 2007, Ultimo, NSW, Australia; School of Life and Environmental Sciences, University of Sydney, 2050, Sydney, NSW, Australia; ARC Centre of Excellence for Translational Photosynthesis, Hawkesbury Institute for the Environment, Western Sydney University, 2753, Sydney, NSW, Australia; Bielefeld University, Germany

**Keywords:** Chlorophyll fluorescence, heat shock, heat shock proteins, metabolomics, raffinose family oligosaccharides, photosynthesis, RNA-Seq, SnRK1, thermotolerance

## Abstract

Understanding how crop varieties acclimate to elevated temperatures is key to priming them for future climates. Here, we imposed a 6 d heat shock treatment (reaching 45 °C) on two genotypes of *Sorghum bicolor* [one sensitive to heat shock (*Sen*) and one tolerant (*Tol*)] growing under two temperature regimes, and carried out a suite of measurements before and during the heat shock. *Sen* consistently reduced photosynthetic functioning during heat shock, while *Tol* increased its photosynthetic rate. Higher abundance of heat shock protein transcripts and metabolites related to heat tolerance were noted for *Tol* when compared with *Sen* both before and during heat shock, which can be attributed to constitutive and inducible responses to elevated temperatures. In addition, important changes in metabolic pathways were clearly identified for *Tol* during heat shock (including up-regulation of raffinose family oligosaccharides and down-regulation of the γ-aminobutyric acid catalytic pathway), even as the concentration of hexose sugars became depleted. We infer *Tol* was able to tolerate elevated temperatures due to up-regulation of osmoprotectants, chaperones, and reactive oxygen species scavengers and by the suppression of SnRK1 via transcripts and metabolites during heat shock. Our results highlight potential targets for attributes of high temperature tolerance that can be utilized in future breeding trials.

## Teaser

Heat shock tolerance within a sorghum genotype was facilitated by up-regulation of osmoprotectants, chaperones, and reactive oxygen species scavengers and by suppression of the metabolic regulatory kinase SnRK1.

## Introduction

With global temperatures constantly increasing, it is now more than ever essential to understand plant responses to elevated temperatures. Over the past 100 years, average temperatures in key agricultural regions have risen by ~1 °C (Zhao *et al.*, 2017), while heatwave probability has up to doubled (Lhotka *et al.*, 2018). These temperature increases are particularly detrimental for agriculture in warmer regions, including key cropping regions such as parts of North America but also countries across Africa where production is not considered high but is essential for feeding local communities (Ortiz-Bobea *et al.*, 2021). C_4_ crops, such as *Zea mays* L. (maize) and *Sorghum bicolor* L. Moench (sorghum) often dominate these hotter climates partly because of their superior water use efficiency (WUE) associated with the presence of a carbon concentrating mechanism (CCM). This results in the CO_2_-saturation of photosynthetic rates and the reduction of photorespiration (Hatch, 1987). This CCM allows C_4_ crops to be highly productive, particularly in warm climates, to the point where a large proportion (~25%) of today’s total plant productivity is generated from this relatively small subset of plant species (~3%) (Still *et al.*, 2003; Beer *et al.*, 2010). In total, C_4_ crops account for over 40% of current world cereal production (FAO, 2019), and so maintaining the high productivity of C_4_ crops is essential to feeding future populations.

Sorghum is the fifth most produced cereal and contributes an essential proportion of cereals to some of the world’s most impoverished countries (Leff *et al.*, 2004). Its superior drought and heat tolerance relative to maize makes it a safer choice in these regions where droughts and heat waves are common (Watson-Lazowski and Ghannoum, 2021). Although heat stress sometimes also results in water stress as a consequence of the elevated water consumption by the crops, many physiological traits, genes, and biochemical pathways involved in the heat and drought responses are unique and independent (Zandalinas *et al.*, 2018; Bhardwaj *et al.*, 2021). Seasonal average temperature optima for crop yield is higher for sorghum than for maize; for example maize requires a minimum of 18 °C, sorghum 25 °C (Hatfield *et al.*, 2011; Lobell and Gourdji, 2012), and optimal temperature for net photosynthesis in sorghum is usually >38 °C, while temperatures above 35 °C negatively impact maize photosynthetic light absorption (Hatfield *et al.*, 2011; Sonawane *et al.*, 2017; Hussain *et al.*, 2019). Hence, studying heat responses in a crop better suited to deal with elevated temperatures may pave the way to a more resilient agriculture and to a reduction in yield losses in a warming world (Lobell and Gourdji, 2012; Hatfield and Prueger, 2015). Furthermore, while optimal temperatures for gas exchange, growth, and yield vary among C_4_ crops, variability within varieties or genotypes can significantly increase the tolerance limit for heat stress. In this regard, substantial genetic variability for grain yield, pollen fertility, seed-set, plant biomass, leaf gas exchange, and biochemistry have been observed in response to high temperatures among sorghum genotypes (Craufurd and Peacock, 1993; Khizzah *et al.*, 1993; Jagtap *et al.*, 1998; Reddy *et al.*, 2011; Djanaguiraman *et al.*, 2014; Singh *et al.*, 2015; Prasad *et al.*, 2021). Current efforts are looking to further increase sorghum productivity as well as integrate sorghum into new regions to help stabilize food supplies (Orr *et al.*, 2020; Hossain *et al.*, 2022; Srivastava *et al.*, 2023). Therefore, knowledge pertaining to heat stress tolerance in this crop is becoming increasingly important.

Heat stress can occur over two time frames, continuous or short term (known as a heat shock), both of which influence plant function at different developmental stages (Wahid *et al.*, 2007). Once temperatures rise above a species’ optimum, it can have negative effects on plant productivity, ranging from reduced photosynthetic rates and reduced yields through to plant death (Barnabás *et al.*, 2008; Schauberger *et al.*, 2017; Yang *et al.*, 2017). Numerous factors can play into responses, including changes in the fluidity of the plasma membrane, misfolded proteins, heightened water loss, alterations in hormone homeostasis, excessive production of reactive oxygen species (ROS) and decreased catalytic activity of key enzymes (Kotak *et al.*, 2007; Barnabás *et al.*, 2008; Qu *et al.*, 2013). Although reproductive structures are commonly the most sensitive components of the plant to heat stress (Hatfield and Prueger, 2015), leaves are also sensitive due to their role in carrying out photosynthesis. Many component processes of photosynthetic metabolism are highly temperature sensitive, although Rubisco deactivation and declines in electron transport rate (ETR) are the critical factors in explaining the decline of net photosynthesis (*A*_n_) above optimal temperatures (Moore *et al.*, 2021; Scafaro *et al.*, 2023). Plants can also display thermal acclimation to optimize carbon gain, including shifting temperature optima of *A*_n_ when growing under different temperature conditions (Yamori *et al.*, 2014; Kumarathunge *et al.*, 2019) and declining mitochondrial respiration at a standard measuring temperature (Atkin and Tjoelker, 2003). Hence, assessing heat tolerance at different growth temperatures can help elucidate a larger range of adaptive and acclimatory mechanisms.

To counteract detrimental effects of high temperatures, plants can use constitutive strategies and inducible responses that involve the expression of genes that encode proteins, such as chaperones and enzymatic ROS scavengers, which are critical for plant thermotolerance (Kotak *et al.*, 2007; Ding *et al.*, 2020). One of the most conserved inducible responses to heat stress is related to a family of proteins known as heat shock proteins (HSPs) (Vierling, 1991; Wang *et al.*, 2004; Waters and Vierling, 2020). These proteins are well documented and conserved across a large range of species (De Maio, 1999). Heat shock factors (HSFs) initiate the transcription of HSPs after the sensing of a range of abiotic stresses (Guo *et al.*, 2016). ROS also play a crucial role in initiating heat stress signalling cascades (Choudhury *et al.*, 2017), and HSFs can be indirectly activated through changes in cellular ROS homeostasis (Driedonks *et al.*, 2015). HSPs play crucial roles in the folding/unfolding of proteins, assembly of multiprotein complexes, transport/sorting of proteins into correct subcellular compartments, cell-cycle control, and signalling, leading to the protection of cells against stress or apoptosis (Li and Srivastava, 2003). Metabolites can also play direct roles in alleviating heat stress by acting as osmoprotectants. These metabolites function to stabilize proteins and membranes during oxidative stress (e.g. trisaccharide raffinose) (Nishizawa *et al.*, 2008). In addition, plants can alter their metabolic pathways to produce beneficial metabolites, such as the well documented γ-aminobutyric acid (GABA) shunt to increase the abundance of GABA. Increased concentrations of GABA can help maintain metabolic homeostasis and reduce the accumulation of H_2_O_2_ generated under heat shock (Bouché *et al.*, 2003).

In this study, our objective was to further elucidate the mechanisms by which *S. bicolor* can tolerate heat shock by exposing two genotypes of *S. bicolor*, with contrasting heat tolerance, to the heat stress that crops often suffer during summer heatwaves. Given the complexity of the plant heat stress response, we investigated multiple growth temperatures and employed a systems approach at the transcriptomic, metabolomic, and physiological levels to elucidate the adaptations and acclimations of the two contrasting *S. bicolor* genotypes. Using information obtained in this study, we begin to elucidate the pathways contributing to both constitutive and inducible responses to heat shock in *S. bicolor*, identifying genes and pathways that can be further investigated via breeding programmes to produce germplasm primed for high-temperature environments and climate-resilient crops.

## Materials and methods

### Plant material

Two genotypes of *Sorghum bicolor* were utilized in this study; FF_SC449-14E and FF_SC906-14E, referred to here as the tolerant (*Tol*) and sensitive (*Sen*) genotype, respectively. The contrasting heat tolerance of these two genotypes was first observed during a prior screening experiment involving several sorghum genotypes and validated during the interaction of elevated CO_2_ concentration with water stress and heat (Al-Salman *et al.*, 2023). Both genotypes have come through the sorghum conversion programme (SCP), which is a backcross breeding scheme in which genomic regions conferring early maturity and dwarfing from an elite donor are introgressed (approx. 4% of genome from the recurrent donor) into exotic sorghum accessions [see Tao *et al.* (2020) and references therein for further information about these lines and the SCP programme].

### Growth conditions and heat shock

Seeds were germinated in trays of Seed and Cutting Potting Mix (Scotts, Australia) on 10 October 2016, planted 3 cm deep, in a controlled cabinet maintained at 25 °C, 60% humidity, and constant darkness. Four days after germination plants were moved into 10 cm deep pots containing a blended soil substrate (soil, sand, and organic material) with added slow-release fertilizer (Osmocote Plus Organic All Purpose, Scotts Pty Ltd, Baulkham Hills, Australia), and placed in a naturally lit, controlled-environment glasshouse (Plexiglas Alltop SDP 16; Evonik Performance Materials, Darmstadt, Germany) at the Hawkesbury Institute for the Environment, Western Sydney University, Richmond, New South Wales Australia (−33.612032, 150.749098). After a week, seedlings of similar size within each genotype (*n*=3) were transplanted into 7.5 litre pots (containing the same soil mix and fertilizer as above) and grown at two thermal conditions. Three plant replicates were arranged in a randomized fashion to minimize any temperature or light gradients in each glasshouse and switched from one glasshouse to the other every 2 weeks. Plants were watered regularly throughout growth.

Plants were then grown under two temperature regimes, with mean daily temperatures during the light period of 22 °C and 35 °C, referred to as low temperature (LT) and high temperature (HT), respectively, and maintained in two adjacent glasshouse bays. There was a daily temperature regulation within each treatment with maximum temperatures around midday (maximum daily temperature of 24 °C and 38 °C for LT and HT, respectively) and a progressive decline through the afternoon. These two contrasting daily temperatures correspond to the range of optimal temperatures for sorghum production documented in the FAO Crop Ecological Requirements Database (ECOCROP; https://gaez.fao.org/pages/ecocrop). A typical diurnal range of ~9 °C and ~13 °C was maintained for LT and HT, respectively, by heating and cooling throughout the day–night cycle (Automated Logic WebCTRL Building Management System; Braemar Th320 Natural gas heater; Dunnair PHS25 Air Conditioner, using Vaisala HMP110 Humidity/Temperature probes and HMT130 Transmitters). Relative humidity was kept close to 60% at all the temperatures (Carel Humidisk 65 humidifier), to ensure vapour pressure deficit did not exceed 1.3 kPa. The photosynthetic photon flux density (PPFD) at canopy height (Apogee Instruments quantum sensor) varied with prevailing weather conditions but was consistent across chambers, and a daytime maximum PPFD ~1500 µmol m^−2^ s^−1^ was regularly observed over the course of the experiment due to clear conditions.

Heat shock was applied to both treatments at 44 d after germination, when plants were still at the vegetative stage and before head formation. This was achieved by raising the air temperature within the glasshouse over 3 d to reach a mean daily temperature of 45 °C on the fourth day, which was then maintained for 6 h d^−1^ and for 3 d (Supplementary Fig. S1).

### Leaf photosynthesis and sampling

Leaf gas exchange was measured twice per plant (*n*=3) for the two genotypes, first at their respective growing conditions (before heat shock 43 d after germination, referred to as B) and then the same plants 1 week later during the heat shock (on the last day of heat shock; referred to as D), using the LI-6400XT infra-red gas analyser (LI-COR Biosciences, Lincoln, NE, USA) with the 2 cm^2^ fluorescence chamber (64-40 leaf chamber fluorometer). The youngest and fully expanded leaf counting from the base of the plants (12–14th leaf depending on growing treatment and genotype) was selected for measurements B and the leaf immediately above for D (as the leaf initially used for gas exchange was sampled for leaf discs). Conditions inside the LI-6400XT chamber were 400 ppm of CO_2_, 2000 μmol m^−2^ s^−1^ light intensity (10% blue light), and the block temperature was set to match the respective growth temperature (24 °C and 38 °C for LT and HT, respectively) for measurements B, and at a common block temperature of 44 °C for measurements D. Measurements of net carbon assimilation rate (*A*_n_), chlorophyll fluorescence [the maximum efficiency of photosystem II (PSII) under the given light conditions, *F*_v_ʹ/*F*_m_ʹ, and the electron transport rate among photosystems (ETR) following Loriaux *et al.* (2013)], and stomatal conductance to water vapour (*g*_sw_) were obtained once gas exchange was stabilized within the cuvette [following the equation of Von Caemmerer and Farquhar (1981)]. Leaf to air temperature change (Δ*T*) is the difference between leaf temperature measured with chamber thermocouple touching underneath the leaf and air temperature measured by the internal air temperature thermistor located beneath the chamber mixing fan. Gas exchange was measured only during clear and sunny days and between 11.00 h and 13.00 h. Dark leaf gas exchange (dark respiration) and dark-adapted chlorophyll fluorescence (the maximum quantum yield of PSII, *F*_v_/*F*_m_) were measured at predawn (04.00 to 05.00 h) the night of the daytime gas exchange measurements. Dark steady state gas exchange with no light, set at 400 ppm of CO_2_ and 25 °C block temperature, was achieved before measurements.

Ten leaf discs of 9.5 mm diameter were collected after the gas exchange measurements from the middle section of the same youngest fully expanded leaf (*n*=3) when plants were 44 d after germination before heat shock (B) and on the last day of the heat shock (D, 51 d post-germination) from the youngest fully expanded leaf immediately above the previously collected (*n*=3), as used for gas exchange. All samples were snap-frozen in liquid nitrogen inside Eppendorf tubes and stored immediately at −80 °C.

### RNA extraction and transcriptomics

For plants grown at 22 °C (LT treatment) RNA sequencing (RNA-Seq) was carried out (*n*=3). Frozen leaf discs (two per biological replicate) were ground into a fine powder using a TissueLyser (Qiagen) in a 2 ml tube with the aid of metal ball bearings. Clamps were frozen at −80 °C before use to ensure the samples remained frozen. Total RNA was extracted using PureZol (Bio-Rad) following the method described in Sharwood *et al.* (2011). Aliquots of RNA were treated with Ambion DNase I (Thermo Fisher Scientific) following the manufacturer’s protocols. Libraries were prepared from the treated RNA using Truseq stranded reagents via the RNase H method (Illumina), followed by BGISEQ DNA nano-ball synthesis in preparation for BGISEQ platform sequencing. Strand specific RNA-seq was carried out on the libraries using a BGISEQ‐500 platform (BGI) with 100 bp paired-end sequencing at BGI Tech Solutions, Hong Kong. Reads were filtered to remove adapter sequences and low-quality reads in-house at BGI Tech Solutions, Hong Kong. Between 75 and 78 million clean reads were returned per sample. These data can be found on GenBank under BioProject accession PRJNA1074961. Reads were aligned to the latest *S. bicolor* transcriptome (McCormick *et al.*, 2018) using the Quasi align mode within Salmon (Patro *et al.*, 2017). This gave a relative abundance measure for each sample [transcripts per million (TPM)]. All TPM values can be found in Supplementary Dataset S1. EdgeR (Robinson *et al.*, 2010) was then used to identify significantly differentially expressed (DE) transcripts (with raw counts as the input) with an inclusion cut-off of 1 count per million in at least three samples. Significant cut-offs used were false discovery rate ≤0.05 and log_2_ fold change >1.5. All genes identified as DE can be found in Supplementary Dataset S2.

### Gene ontology and orthologue identification

To assess functional enrichment of the DE transcripts, gene ontology (GO) over-representation categories were assessed using gProfiler using the default settings (Raudvere *et al.*, 2019). Enrichment analysis used the Ensemble Plants *S. bicolor* NCBI v3 assembly as a reference for GO representation. For genotype comparisons, GO enrichment was carried out on all DE transcripts. For response to heat shock, GO enrichment was carried out on only the DE transcripts that were uniquely up- or down-regulated in either *Tol* or *Sen* in response to heat shock. All GO categories identified as being over-represented can be found in Supplementary Dataset S2. Genes of interest were extracted from published literature and the *S. bicolor* orthologues identified using BLASTp (Supplementary Dataset S3).

### NanoString

For plants grown at 22 °C and 35 °C (LT and HT, respectively) NanoString was used to calculate gene expression of key genes. Genes of interest (based on previous heat stress literature) and reference gene (based on RNA-seq transcript expression) sequences were identified within the *S. bicolor* v3.1 transcriptome (McCormick *et al.*, 2018) and quantified using the NanoString nCounter Expression analysis system (NanoString Technologies, USA) following Kim *et al.* (2018). Briefly, 200 ng of purified RNA (extracted as above) was hybridized to the corresponding nCounter Reporter and Capture probes (Supplementary Dataset S4), along with hybridization buffer for a period of 16 h in a Veriti 96-well thermal cycler (Thermo Fisher Scientific) set to 65 °C. An nCounter Prep Station and nCounter Digital Analyzer (NanoString Technologies) was used to prepare the sample cartridge and count the individual fluorescent barcodes associated with each gene of interest, respectively. A maximum resolution scan encompassing 555 fields of view was performed and the data were processed using nSolver 4.0 (NanoString Technologies) to identify statistically significant differences. Normalization of gene counts was performed using the three best reference genes as identified by nCounter Advanced Analysis 2.0 via the geNorm algorithm (Vandesompele *et al.*, 2002).

### Metabolomics

From plants grown at 22 °C (LT) leaf metabolomic analyses were carried out for the two genotypes before and during the heat shock (*n*=3). Polar metabolites were extracted from four leaf discs by first grinding in a mortar and pestle with a monophasic mixture of methanol–chloroform–water, then subsequently phase separating by adding additional water and chloroform. The aqueous phase with a final volume of 1100 µl was used for subsequent metabolomic analyses.

Capillary electrophoresis–mass spectrometry (CE-MS) was used for untargeted profiling of organic N monomers in the aqueous fraction of methanol–chloroform–water extracts, as described previously (Warren *et al.*, 2011, 2012; Warren, 2013). Extracts were concentrated 8-fold by evaporating under reduced pressure (Vacufuge, Eppendorf) then made up in 100 mM ammonium formate (pH 9.5) in 25% (v/v) acetonitrile that contained two instrumental internal standards. CE-MS was performed with a capillary electrophoresis system (P/ACE MDQ, Beckman-Coulter, Fullerton, CA, USA) equipped with a bare fused silica capillary (50 µm i.d. × 100 cm long) interfaced via a co-axial sheath-flow sprayer (G1607A, Agilent, Waldbronn, Germany) with 4 µl min^−1^ sheath liquid (50% methanol with 0.1% formic acid) to a mass spectrometer (AmaZon SL, Bruker Daltonics, Bremen, Germany). Samples were injected at 3 psi for 30 s and separated with an electrolyte of 2 M formic acid with 20% (v/v) methanol under 30 kV positive polarity. The mass spectrometer was set to scan at 8100 Da s^−1^ from 50 to 255 Da. Compounds were identified and quantified based on comparison of migration times, [M+H]^+^, MS^2^ and (for some compounds) MS^3^ with 63 authentic standards run under the same conditions on the same instrument, as described previously (Warren, 2013).

Gas chromatography–mass spectrometry (GC-MS) was used for untargeted profiling of methoximated trimethylsilyl derivatives of organic acids, sugars, and sugar alcohols in the aqueous fraction of methanol–chloroform–water extracts, as described previously (Lisec *et al.*, 2006). A 50 μl aliquot of 0.02 mg ml^−1^ ribitol (internal standard) was added to 200 μl of extract dried under reduced pressure (Vacufuge, Eppendorf). Dried samples were derivatized first with 40 μl of 20 mg ml^−1^ methoxyamine hydrochloride in pyridine (90 min at 37 °C) then with 70 μl of *N*-methyl-*N*-trifluoroacetamide with 1% trimethylchlorosilane (30 min at 37 °C). A 1 μl sample was splitless-injected into an injection port liner (FocusLiner, SGE, Ringwood, Australia) at 250 °C and separated by capillary gas chromatography on an arylene-modified 5% diphenyl–95% dimethyl polysiloxane stationary phase (30 m long×0.25 mm ID×0.25 μm film thickness with a 10 m ‘guard column’; Rxi-5SilMS, Restek, Bellfonte, PA, USA). The column was held at 70 °C for 3 min, raised to 330 °C at 6 °C min^−1^, and then held at 330 °C for 10 min. Helium (99.999%, BOC, North Ryde, NSW, Australia) was used as the carrier gas at a constant flow of 1 ml min^−1^. The transfer line was held at 280 °C and the ion source at 250 °C. The column eluent was ionized by electron impact (70 eV) and mass spectra were collected from 70 to 600 amu at 6.67 scans s^−1^ (GCMS-QP2010Plus, Shimadzu, Kyoto, Japan). Metabolites were identified by comparing retention indices and mass spectra with a laboratory mass spectral/retention index library based on 130 chemical standards plus the Golm Metabolome Database (Schauer *et al.*, 2005), Agilent Fiehn, and NIST libraries.

For metabolites measured on both analytical platforms, only the most reliable measurements were reported (e.g. CE-MS for amino acids). For metabolites with multiple derivatives, data were de-replicated to a single peak. This pre-processing step involved either an averaging of the multiple peaks per metabolite (in cases where peak areas were similar), or deletion of those peaks that were deemed less reliable (e.g. low abundance peaks). Metabolites that were not present in ≥2 samples were omitted from the analysis.

### Statistics

Either a two-way (genotype, heat shock) or a three-way (genotype, heat shock, growth temperature) ANOVA was utilized within R (R CoreTeam, 2013) to identify significant changes in metabolite content and physiological traits, respectively. Each variable was first tested for normality using the Shapiro–Wilk test before analysis, then, if necessary, transformed using the log function in R. If there was a significant value returned (*P*≤0.05), Tukey’s post-hoc test was used to indicate which groups differed significantly. Principal component analyses (PCA) were carried out in R using the prcomp function and then plotted using the ggbiplot package (R CoreTeam, 2013). Data was centred and scaled prior to PCA analysis. A cut-off of ≥1 TPM for each sample was used for a gene to be included in the PCA. The two orthogonal principal components presented account for the largest percentage of variation within the dataset, and are therefore assumed to be the most important components.

## Results

### Heat tolerant sorghum genotype (*Tol*) maintains physiological functioning during an applied heat shock regardless of growth temperature

**Fig. 1.**
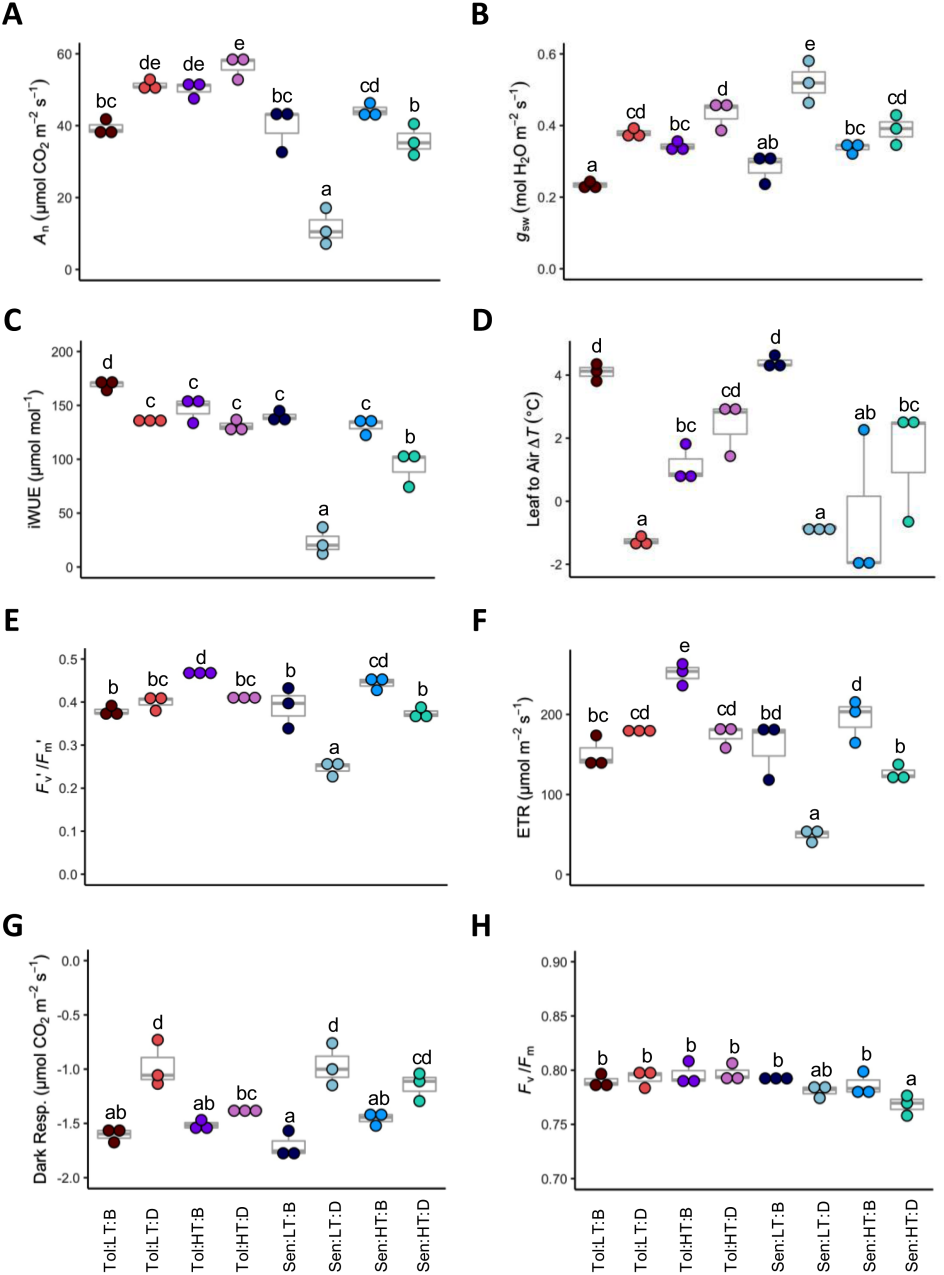
Physiological responses to heat shock. Measurements of (A) assimilation (*A*_n_), (B) stomatal conductance (*g*_sw_), (C) intrinsic water use efficiency (iWUE), (D) leaf to air temperature change (Δ*T*), (E) *F*_v_ʹ/*F*_m_ʹ, (F) electron transport rate (ETR), (G) dark respiration, and (H) *F*_v_/*F*_m_. Measurement temperatures are described in ‘Materials and methods’. Each dot represents a biological replicate (*n*=3), and the box and whisker plots illustrate the median, upper quartile, and lower quartile. Where two groups do not share a letter, a significant difference (*P*≤0.05) was identified, determined via a three-way ANOVA and Tukey’s post-hoc test. *Sen*, sensitive genotype; *Tol*, tolerant genotype; LT, low temperature (22 °C); HT, high temperature (35 °C); B, before heat shock; D, during heat shock.

The two genotypes of *Sorghum bicolor* investigated (*Tol* and *Sen*) showed contrasting physiological responses to exposure to a 45 °C heat shock when grown at either 22 °C or 35 °C (LT and HT, respectively). When grown at LT, a significant reduction (*P*≤0.05) in assimilation rate (*A*_n_), maximum efficiency of PSII under the given light or in darkness (*F*_v_ʹ/*F*_m_ʹ and *F*_v_/*F*_m_), and quantum yield of photosystem II (Φ_PSII_) that was reflected in the ETR was identified in *Sen* in response to heat shock (Fig. 1). On the other hand, *Tol* maintained all photosynthetic parameters during the heat shock, and even significantly increased *A*_n_ (*P*≤0.05) (Fig. 1A) and showed continued growth (Supplementary Fig. S2). When grown at HT, again, *Tol* maintained physiological functioning during heat shock and *Sen* exhibited a significantly reduced (*P*≤0.05) assimilation rate, albeit to a significantly lesser extent (*P*≤0.05) than *Sen* grown at LT (Fig. 1). Leaf temperature reached 28 °C for LT and 41.5 °C for HT before heat shock, and slightly below 44 °C during the heat shock, for both genotypes. Temperature difference between the leaf and the air (leaf to air Δ*T*) was consistent between the two genotypes when grown at LT (Fig.1D). Leaves were ~4 °C warmer than air when growing at LT and ~1 °C colder during the heat shock. Less variation was apparent in leaf to air Δ*T* when grown at HT (Fig. 1D). Both genotypes had higher stomatal conductance during heat shock; however, *Sen* required significantly (*P*≤0.05) more open stomata than *Tol* during heat shock to achieve the same leaf temperature (Fig. 1B). Both genotypes showed significant thermal acclimation (lower rates) of dark respiration to heat shock (Fig. 1G), measured at predawn at a common temperature of 25 °C. These results indicate *Tol* can maintain or even improve photosynthetic functioning during heat shock at 45 °C, while photosynthetic functioning is either inhibited or negatively regulated in *Sen*. As the largest contrast between the *Tol* and *Sen* genotypes was seen at LT, this treatment was chosen for more in-depth analysis to further elucidate the underpinning mechanisms behind this genotype specific heat tolerance.

### Distinct constitutive and inducible metabolite profiles of *Tol* and *Sen* genotypes highlight metabolites of interest related to heat tolerance in sorghum

**Fig. 2.**
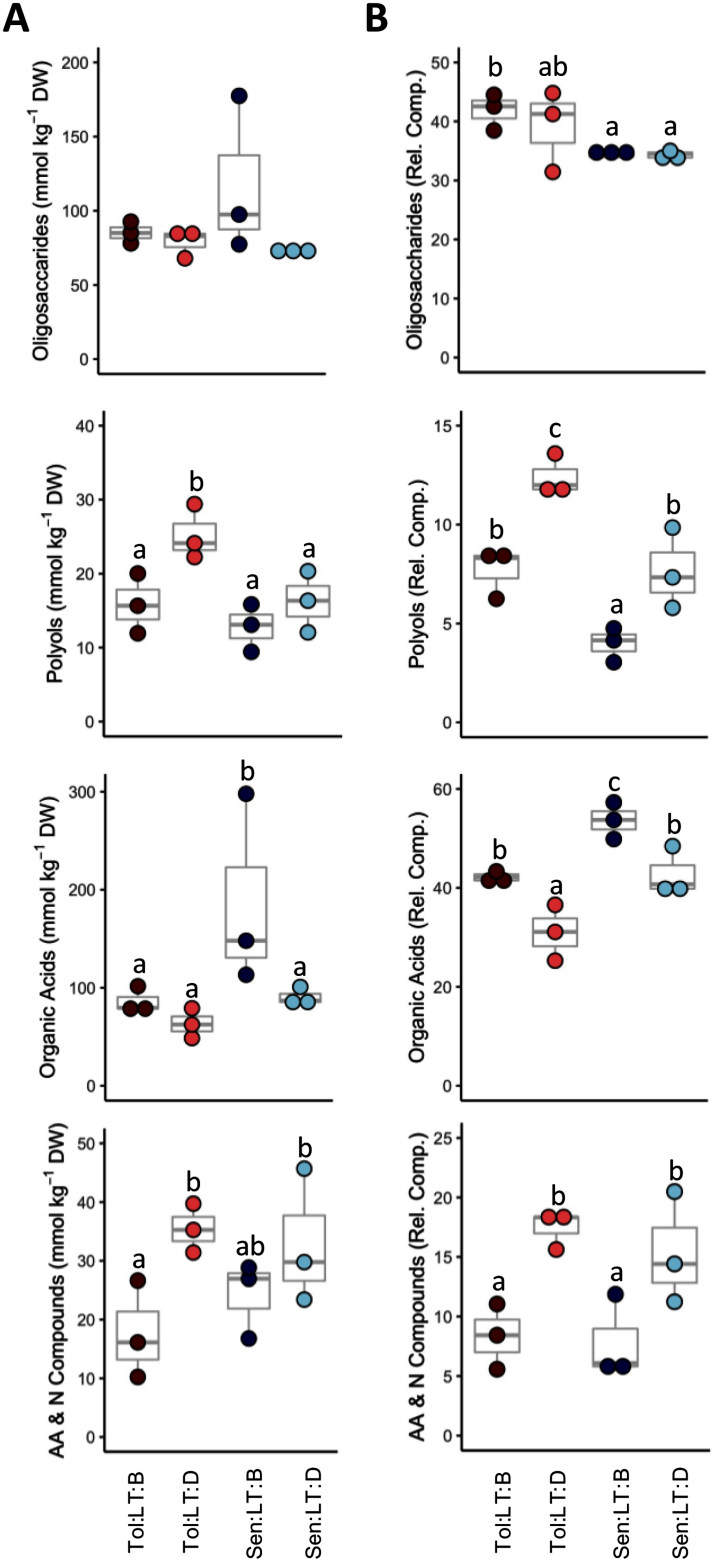
Response of major biochemical pools to heat shock. Soluble organic compounds grouped into four main biochemical pools and expressed by concentration relative to dry leaf matter (A) or relative to total polar organic metabolites (B). The panels show oligosaccharides (fructose, galactose, gentiobiose, glucose, mannose, raffinose, and sucrose), polyols (*allo*-inositol, galactinol, galactosylglycerol, glycerol, maltitol, mannitol and *myo*-inositol), organic acids [2-oxoglutaric acid, benzoic acid, caffeic acid, caffeoyl-quinic acid (3 *trans*), *cis*-aconitic acid, citric acid, coumaroyl-quinic acid (3 *cis*), coumaroyl-quinic acid (3 *trans*), dehydroascorbic acid, erthythronic acid, fumaric acid, galactaric acid, galactonic acid, glucaric acid, gluconic acid, glucono-1,5-lactone, glyceric acid, maleic acid, malic acid, nicotinic acid, quinic acid, shikimic acid, succinic acid, and threonic acid], and amino acids and N compounds (amino acids, amines, amides and quaternary ammonium derivatives—ethanolamine, choline, ornithine, lysine, arginine, trimethyllysine, histidine, butyramide, β-alanine, γ-aminobutyric acid, glycine, alanine, alanine–alanine, serine, valine, isoleucine, leucine, trigonelline, asparagine, threonine, proline, methionine, glutamine, betaine, glutamic acid, phenylalanine, tryptophan, citrulline, aspartic acid, and tyrosine). These metabolites accounted for over 90% of the measured organic soluble metabolites in sorghum leaves. Each dot represents a biological replicate (*n*=3), and the box and whisker plots illustrate the median, upper quartile, and lower quartile. Where two groups do not share a letter, a significant difference (*P*≤0.05) was identified, determined via a two-way ANOVA and Tukey’s post-hoc test. *Sen*, sensitive genotype; *Tol*, tolerant genotype; LT, low temperature (22 °C); B, before heat shock; D, during heat shock.

A total of 83 metabolites were measured using GC-MS and CE-MS both before and during heat shock for both genotypes grown at LT (Supplementary Dataset S5). Soluble organic compounds were dominated by oligosaccharides and organic acids before heat shock in both genotypes, with a significantly (*P*≤0.05) larger fraction of organic acids present in *Sen*, and a significantly larger fraction of oligosaccharides present in *Tol* (Fig. 2). Heat shock significantly increased the absolute concentration of polyols, amino acids, amides, and quaternary ammonium derivatives in *Tol* (Fig. 2A), although the polyols and amino acids fraction also increased in *Sen* during heat shock when expressed as a relative fraction of total soluble organic metabolites (Fig. 2B). On the other hand, heat shock reduced the fraction of organic acids in both genotypes. Metabolites that had a significant (*P*≤0.05) genotype, heat shock, or interaction response were identified (Supplementary Dataset S5). In total, 17 metabolites exhibited a significant genotype response (*P*≤0.05). Two of these metabolites (trimethylamine-*N*-oxide and *myo*-inositol) had significantly higher concentrations in *Tol* compared with *Sen* and have previously been positively linked to abiotic stress tolerance (Mattoo *et al.*, 2015; Zhai *et al.*, 2016; Catalá *et al.*, 2021).

**Fig. 3.**
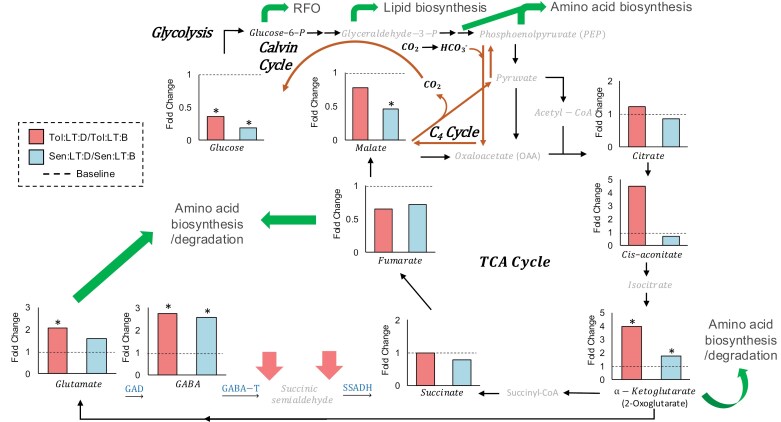
Regulation of the TCA cycle in response to heat shock. Changes in the concentration of metabolites linked to the TCA cycle and amino acid biosynthesis as a consequence of heat shock. Bars represent fold change in the metabolite concentration (calculated using concentrations relative to dry leaf matter) before and during heat shock for each genotype (*n*=3). Dashed lines on each graph represent the baseline fold change of 1. Significant differences in response to heat shock were determined by a two-way ANOVA and Tukey’s post-hoc test. An asterisk indicates a significant difference (*P*≤0.05) between the means (Supplementary Dataset S5). Metabolites written in grey were not detected. Green arrows represent suggested up-regulated pathways and red arrows down-regulated reactions. *Sen*, sensitive genotype; *Tol*, tolerant genotype; LT, low temperature (22 °C); HT, high temperature (35 °C); B, before heat shock; D, during heat shock; GABA-T, 4-aminobutyrate transaminase; GAD, glutamic acid decarboxylase; SSADH, succinic semialdehyde dehydrogenase.

The abundance of several amino acids derived from oxaloacetate and pyruvate (e.g. asparagine, threonine, alanine, and valine) was significantly up-regulated (*P*≤0.05) in response to heat shock in both genotypes (Supplementary Dataset S5). In contrast, the abundance of two oxaloacetate precursors from the TCA cycle (fumarate and malate) were reduced in response to heat shock in *Sen* (Fig. 3). On the other side of the TCA cycle, heat shock significantly increased the concentration of 2-oxoglutarate in both genotypes, which correlated with a significant increase of glutamate in *Tol* and GABA in both genotypes (Fig. 3).

**Fig. 4.**
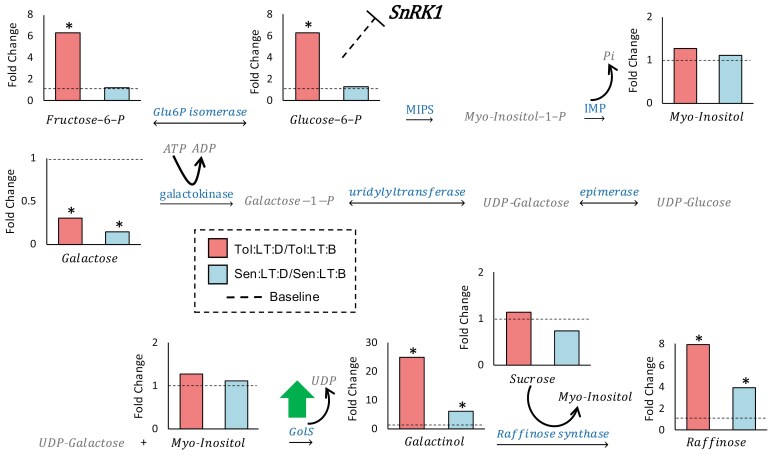
Regulation of raffinose family oligosaccharide (RFO) biosynthesis and related pathways in response to heat shock. Changes in the concentration of metabolites linked to RFO biosynthesis, including the biosynthesis of *myo*-inositol and inhibition of sucrose non-fermenting-1-related protein kinase 1 (SnRK1). Bars represent fold change in the metabolite concentration (calculated using concentrations relative to dry leaf matter) before and during heat shock for each genotype (*n*=3). Dashed lines on each graph represent the baseline fold change of 1. Significant differences in response to heat shock were determined by a two-way ANOVA and Tukey’s post-hoc test. An asterisk indicates a significant difference (*P*≤0.05) between the means (Supplementary Dataset S5). Metabolites written in grey were not detected. Green arrows represent suggested up-regulated reactions. *Sen*, sensitive genotype; *Tol*, tolerant genotype; LT, low temperature (22 °C); HT, high temperature (35 °C); B, before heat shock; D, during heat shock; GolS, galactinol synthase; IMP, inositol-1-phosphate phosphatase; MIPS, d-*myo*-inositol 3-phosphate synthase.

Several key sugar molecules (glucose, galactose, mannitol, and fructose) were significantly down-regulated (*P*≤0.05) in response to heat shock in both genotypes. However, the trisaccharide raffinose (a known osmoprotectant) significantly increased (*P*≤0.05) in abundance in response to heat shock in both genotypes (Fig. 4). In addition, glucose-6-phosphate (G6P) exhibited a significant interaction (genotype×heat shock; *P*≤0.05), with only *Tol* accumulating G6P in response to heat shock (Fig. 4).

Additional metabolites that had a significant interaction (genotype×heat shock; *P*≤0.05) provide further insight into how the two genotypes differ in their response to heat shock (Supplementary Dataset S5). Ethanolamine (2-aminoethanol), the second most abundant head group for phospholipids and a component in the formation of cellular membranes, was significantly enhanced (4-fold) in *Tol* during heat shock, while no change was observed in *Sen*. Ethanolamine is biosynthesized by decarboxylation of serine, which also showed up-regulation in *Tol*, but not in *Sen*. Significantly enhanced levels of galactinol [an additional member of the raffinose family oligosaccharides (RFO)] was apparent in both genotypes in response to heat shock, but to a greater extent in *Tol*, and may again be indicative of an improved osmoprotection capacity within *Tol* (Panikulangara *et al.*, 2004).

### Transcriptome of *Tol* is enriched in abiotic stress response genes

**Fig. 5.**
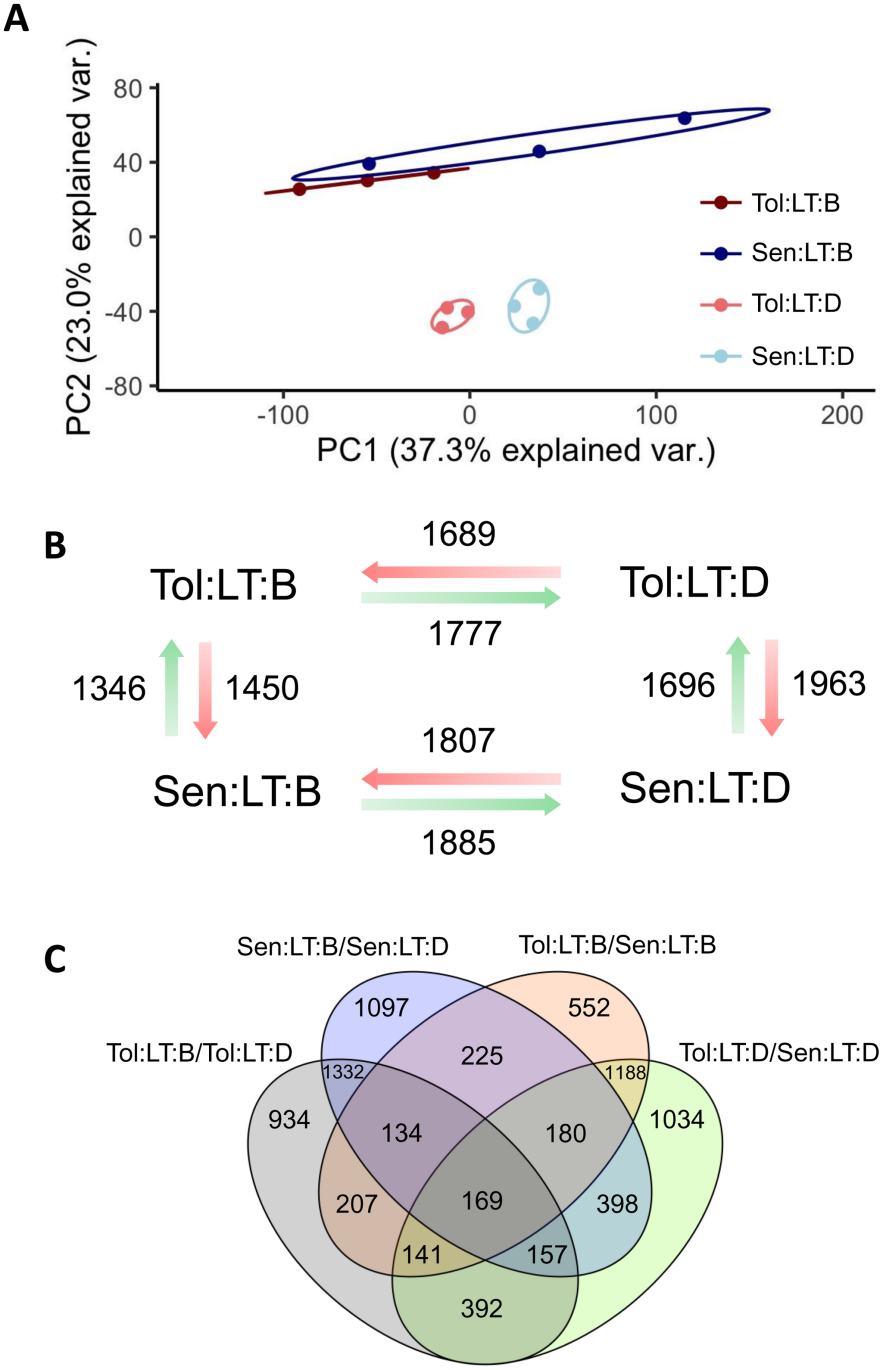
Transcriptomic responses to heat shock. (A) Principal component analysis of the transcriptomic profile for each sample. The two axes correspond to the two principal components that account for the largest percentage of variation within the dataset. Transcripts per million (TPM) values were used as the input and a cut-off of ≥1 TPM for each sample was used for a gene to be included in the PCA. (B) The number of significantly differentially expressed (DE) transcripts (false discovery rate ≤0.05) for comparison between genotypes and in response to heat stress. Numbers by green and red arrows signify the number of up-regulated and down-regulated DE transcripts, respectively. (C) A Venn diagram illustrating the overlap of the DE transcripts for each comparison made. Where ovals overlap, the number stated corresponds to the number of DE genes shared by the corresponding comparisons. For this analysis, up- and down-regulated DE transcripts were combined. *Sen*, sensitive genotype; *Tol*, tolerant genotype; LT, low temperature (22 °C); B, before heat shock; D, during heat shock.

Transcriptomic analyses, via RNA-Seq, were carried out on both genotypes grown at LT both before and during heat shock. PCA, carried out on the TPM values generated for each gene, showed *Tol* and *Sen* had distinct transcriptomic profiles during heat shock (Fig. 5A). A similar number of significantly DE transcripts (false discovery rate ≤0.05) were identified both between genotypes and in response to heat shock (Fig. 5B). The overlap of the DE transcripts highlighted sets of genes that were DE in multiple comparisons, and there were many genes that appear conserved in their response to heat shock within the two genotypes (Fig. 5C). *Rubisco activase-α* (*Rca-α*; Sobic.005G231600), a photosynthetic gene known to respond to high temperature in C_4_ grasses, was significantly up-regulated in both genotypes in response to heat shock (Supplementary Dataset S2). There were also large numbers of DE transcripts that were unique to either genotype, both before and during heat shock, as well as in response to heat shock (Fig. 5C). For example, the alternative Rca isoform *Rca-β* (Sobic.005G231500), had significantly elevated expression in *Tol* when compared with *Sen* both before and during heat shock (Supplementary Dataset S2).

GO was used to identify functional over-representation in the identified DE transcripts. Before heat shock, the DE transcripts that were up-regulated in *Tol* when compared with *Sen* were enriched in numerous abiotic stress response categories, including GO categories specifically related to heat stress (Supplementary Dataset S2). This suggests *Tol* may be primed to respond to heat shock. During heat shock, numerous categories related to metabolic processes were up-regulated in *Tol* when compared with *Sen* (Supplementary Dataset S2). DE transcripts that were uniquely up and down-regulated in response to heat shock were also identified by removing those that were common to both genotypes. For those unique to *Tol*’s response to heat shock, again, enrichment in GO categories related to metabolic processes were identified within the up-regulated DE transcripts (Supplementary Dataset S2). These results suggest *Tol* up-regulates metabolic processes in response to heat shock, a response not apparent for *Sen*.

### Elevated heat shock protein transcript abundance primes *Tol* to respond to heat shock

**Fig. 6.**
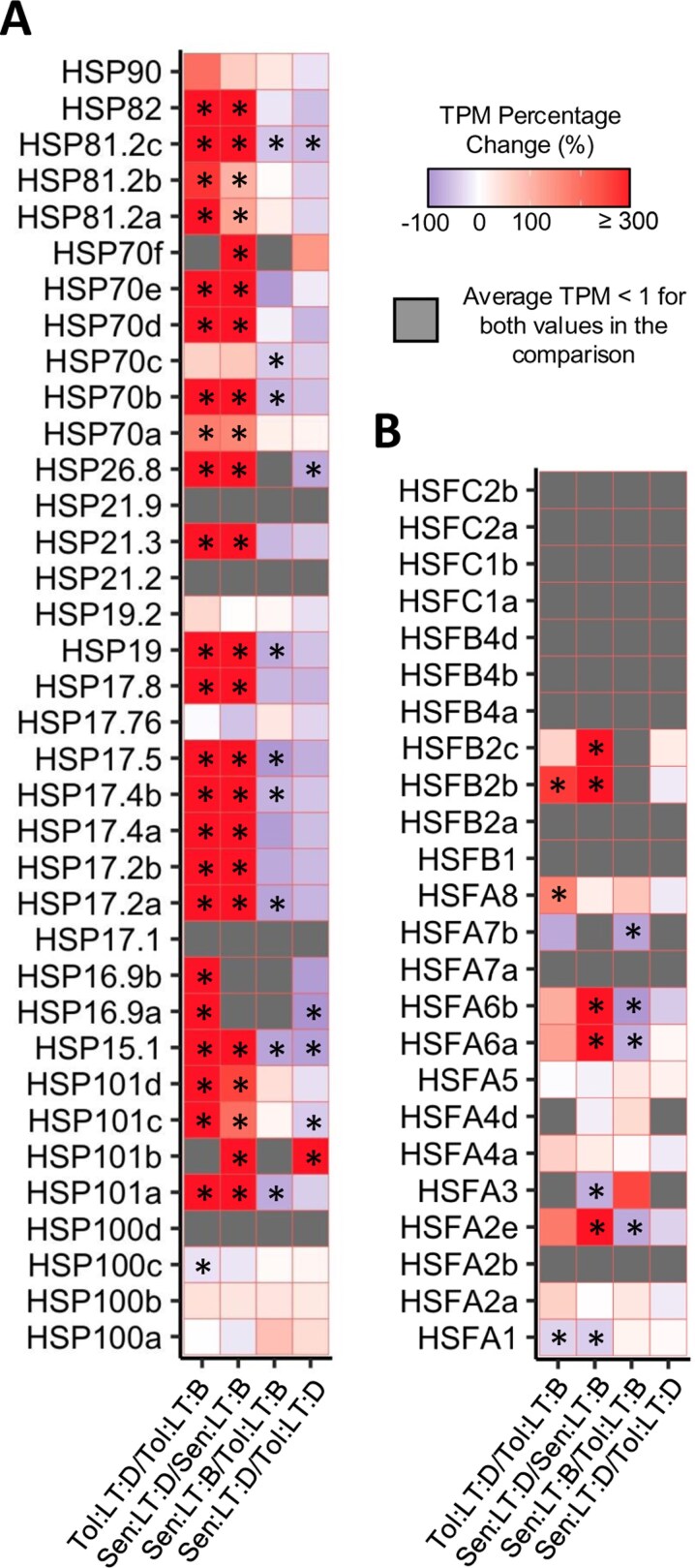
Transcript abundance changes in heat shock factors and proteins. Transcript per million (TPM) percentage change of known heat shock protein (HSP) genes (A) and heat shock factor (HSF) genes (B). Group comparisons shown as *B*/*A*, with percentage change calculated as [(*B*−*A*)/*A*]×100. Asterisks highlight where a transcript was significantly differentially expressed (false discovery rate ≤0.05) for the corresponding comparison. A list of the corresponding *S. bicolor* accession numbers for each gene can be found in Supplementary Dataset S3. *Sen*, sensitive genotype; *Tol*, tolerant genotype; LT, low temperature (22 °C); B, before heat shock; D, during heat shock.

The transcript abundance of HSPs drastically increased in response to heat shock in both genotypes when grown at LT (Fig. 6A). However, expression of several HSP transcripts were genotype specific, and *Tol* had significantly greater transcript abundance of numerous HSPs both before and during heat shock, including isoforms of HSP15 and HSP81 (Fig. 6A). Less drastic changes in transcript expression were seen for HSFs, with a large number showing no significant change or not being expressed above 1 TPM (Fig. 6B). Nevertheless, several HSFs had significantly higher expression during heat shock (compared with before heat shock) within *Sen*. Interestingly, this set of HSFs also had significantly higher transcript expression in *Tol*, when compared with *Sen*, prior to heat shock. Additional HSF targets that are related to the patterns of metabolism seen in Fig. 4 are noted in Supplementary Dataset S2. For example, galactinol synthase 1 (*GolS1*; Sobic.001G391300) was significantly up-regulated in only *Tol* in response to heat shock and has been shown to be a HSF target (Panikulangara *et al.*, 2004). As HSPs play such a crucial role in the response to heat, NanoString was used to support the transcript expression of each HSP within plants grown at LT, as well as shed light on HSP expression for plants grown at HT (Supplementary Fig. S3). Trends noted via RNA-Seq largely matched those seen via NanoString for plants grown at LT, supporting the expression patterns noted in Fig. 6.

### Sustained photosynthetic functioning in *Tol* is associated with increased metabolism during heat shock

Although the concentrations of key carbohydrates were reduced in both genotypes in response to heat shock, the total concentration of soluble metabolites measured during heat shock was unchanged in *Tol* but decreased to 66% in *Sen* (Supplementary Dataset S5), and the abundance of transcripts related to several aspects of metabolism were up-regulated in *Tol* (Supplementary Dataset S2). For example, amide, arginine, and small molecule metabolism related GO categories were identified as up-regulated in *Tol* only. Glutamate concentration was significantly enhanced in *Tol* during heat shock, glutamate being a metabolite that acts as a precursor to other protein amino acids (such as proline, arginine, and cysteine), non-protein amino acids (GABA), antioxidants (e.g. glutathione), and polyamines.

**Fig. 7.**
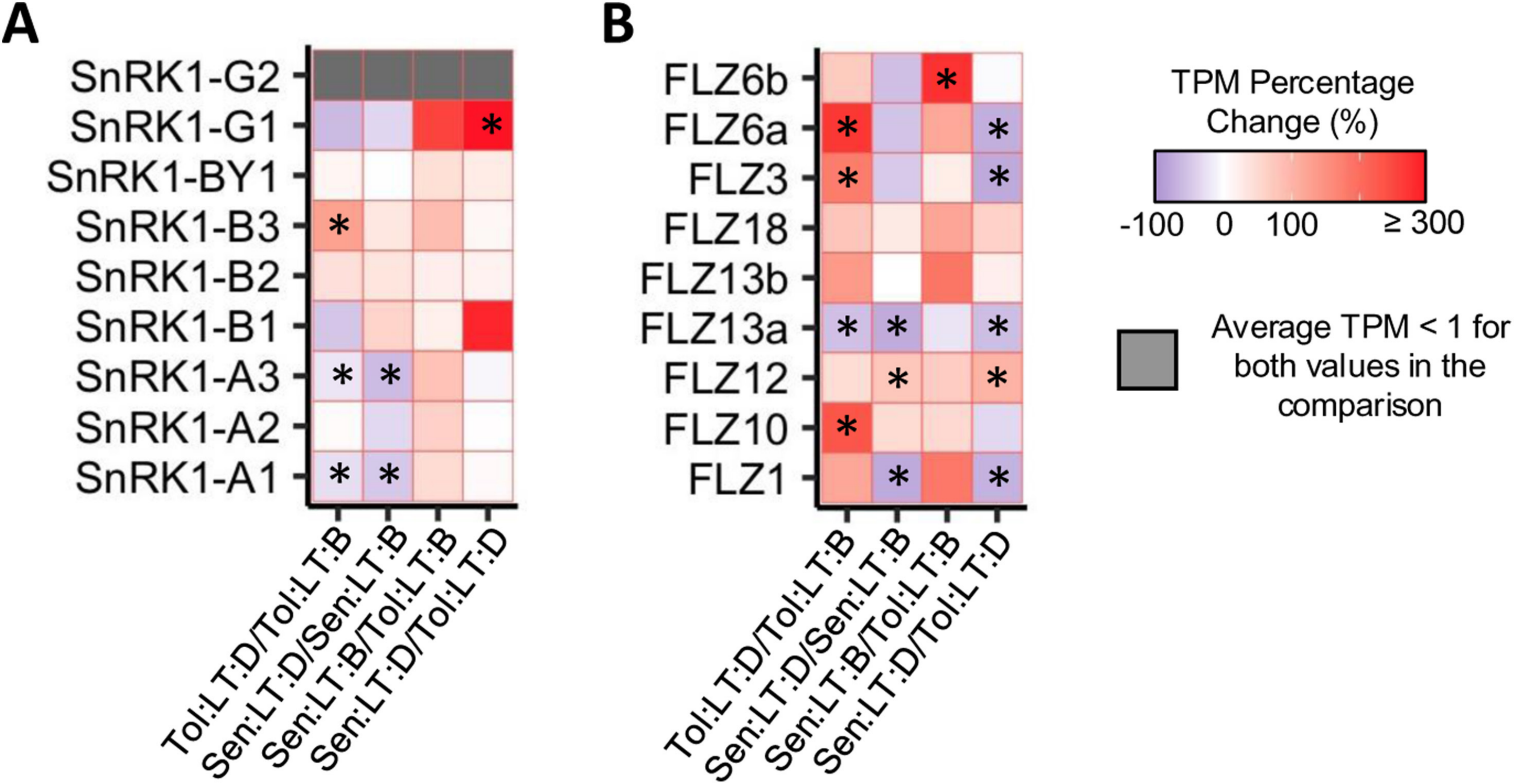
Transcript abundance changes in SnRK1 components and FLZ genes. Transcript per million (TPM) percentage change of known components of SnRK1 (A) and known FCS-Like Zinc Finger (FLZ) genes (B). Group comparisons shown as *B*/*A*, with percentage change calculated as [(*B*−*A*)/*A*]×100. Black asterisks highlight where a transcript was significantly differentially expressed (false discovery rate ≤0.05) for the corresponding comparison. A list of the corresponding *S. bicolor* accession numbers for each gene can be found in Supplementary Dataset S3. *Sen*, sensitive genotype; *Tol*, tolerant genotype; LT, low temperature (22 °C); B, before heat shock; D, during heat shock.

Minimal genotype specific changes in transcript expression were identified for the individual components of the SnRK1 complex, a central regulator of cellular metabolism (Fig. 7A). However, several transcripts have been identified that can inhibit components of the SnRK1 complex, including genes from the FCS-like zinc finger (FLZ) family. The expression of all FLZ transcripts was extracted (Fig. 7B), and several had significantly increased transcript expression (in response to heat shock) in *Tol* only. These transcripts corresponded to FLZ genes previously linked to SnRK1 suppression (*FLZ3*, *FLZ6*, and *FLZ10*) (Jamsheer *et al.*, 2018; Bortlik *et al.*, 2024), indicating a suppression of SnRK1 may be required to facilitate continued metabolism in *Tol* during heat shock, as key sugars become depleted. G6P has also been shown to inhibit SnRK1, and an increase in G6P abundance in response to heat shock was seen only in *Tol* (Fig. 4; Supplementary Dataset S5), strengthening the evidence that inhibition of SnRK1 may be occurring in *Tol* to maintain metabolism during heat shock.

### Constitutive and inducible responses promote tolerance to heat shock in *Tol*

**Fig. 8.**
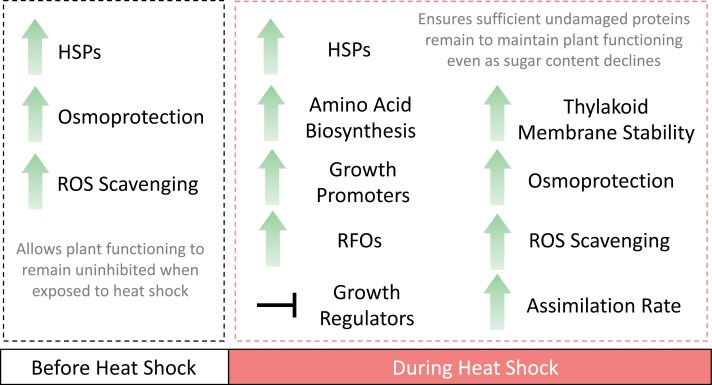
Overview of the response of *Tol* to heat shock. The acclimations and adaptations, both before and during heat shock, that underpin the response of the heat tolerant genotype (*Tol*) of *S. bicolor* to heat shock. Green arrows represent increases noted in this study when comparing *Tol* with the heat sensitive genotype (*Sen*) prior to heat shock, and both to *Sen* and in response to heat shock during heat shock. HSPs, heat shock proteins; RFOs, raffinose family oligosaccharides; ROS, reactive oxygen species.

By taking a multi-omics approach we highlight how genotypes within a single species can differ in their response to heat shock. Further to this, due to one genotype exhibiting a tolerant response to heat shock, we can summarize the adaptations and acclimations that occur specifically in said genotype (Fig. 8).

## Discussion

As temperatures rise across the world, crop yields will be negatively affected unless action is taken (Fahad *et al.*, 2017). Heat tolerance involves many independent traits, but discriminating the confounding effects during breeding evaluation and selection is challenging (Prasad *et al.*, 2021). If we wish to prepare crops for future climates, we need to first comprehensively understand responses to heat stress, and especially how heat tolerant genotypes function both before and during exposure to elevated temperatures. Here, we exposed to a controlled heat shock two genotypes of *Sorghum bicolor* grown at multiple growth temperatures, one of which is tolerant (*Tol*) and the other sensitive (*Sen*) to elevated temperature. By identifying and comparing the responses of the two genotypes we highlight and connect specific traits that correspond to heat tolerance within *S. bicolor*, furthering our understanding of how plants respond to short-term heat shock.

### *Tol* retains photosynthetic functioning during heat shock

It would be expected that a heat shock reaching 45 °C sustained for several hours a day (and over 3 d) would induce a detrimental reduction in the net photosynthesis of *S. bicolor*, due to this temperature being above previously reported optimum temperatures for *S. bicolor* (Hammer *et al.*, 1993; Yan *et al.*, 2011; Sonawane *et al.*, 2017). However, here, *Tol* was able to avoid any reductions in photosynthetic rate (*A*_n_) for the duration of the heat shock, regardless of growth temperature (Fig. 1). Although enzyme activities were not measured, the increase in *A*_n_ measured in *Tol* during the heat shock suggests there was no enzyme deactivation up to 45 °C. This is in contrast to the often-observed inactivation of Rubisco at temperatures above 30 °C in C_4_ crops (Crafts-Brandner and Salvucci, 2002; Perdomo *et al*., 2017), and suggests that Rubisco activase (Rca) was not inactivated in *Tol* at temperatures up to 45 °C. Similar results have been noted in the C_4_ species *Setaria viridis*, where little change in photosynthetic rate was seen in plants grown at 42 °C (Zhang *et al*., 2023, Preprint). The mutual increase and positive correlation between *A*_n_ and the rate of linear electron transport (ETR) in *Tol*, and the decrease observed in *Sen*, highlight the importance of maintaining electron transport functionality under heat shock for the thermal acclimation of photosynthesis. A decline in the rate of accepting electrons from PSII leads to PSII reaction centres being overly oxidized (P680^+^) under high temperature (noted by the *F*_v_ʹ/*F*_m_ʹ reduction in *Sen*, but not in *Tol*), promoting ROS formation. This can damage PSII D1 protein *de novo* synthesis, damaging thylakoid membranes and promoting leakage of protons from thylakoids, which further diminishes the by-products of linear electron transport (ATP and NADPH) (Bukhov *et al.*, 1999; Takahashi and Murata, 2008; Zhang and Sharkey, 2009). The maintenance of *F*_v_/*F*_m_ at predawn indicated that despite the large reduction of *A*_n_ and ETR during the day in *Sen*, there was no permanent photodamage to the leaves in either genotype.

Stomatal conductance (*g*_sw_) significantly increased in both genotypes, a common response to elevated temperatures as the plant attempts to cool the leaf via transpiration (Farquhar and Sharkey, 1982; Urban *et al.*, 2017; Al-Salman *et al.*, 2023). In fact, higher *g*_sw_ during the heat shock led to higher leaf transpiration and negative leaf-to-air temperature difference (Δ*T*) in LT plants during heat shock, a strategy to cool down the leaf observed in other grass species (Scafaro *et al.*, 2016). However, *Sen* showed significantly larger increase in *g*_sw_ than *Tol* during heat shock in 22 °C (LT)-grown plants, likely due to the greater heat shock sensitivity of photosynthesis in *Sen*. Under field conditions, the capacity to cool down the leaf by heat loss due to evapotranspiration is also strongly affected by boundary layer conductance acting in series with the stomatal conductance. *Sen* has wider leaves than *Tol* (Al-Salman *et al.*, 2023) and hence lower boundary conductance, and thus we can venture that the increased aperture of stomata experienced by *Sen* is needed to compensate for the higher resistance to water evaporation in the wider-leaf genotype, as observed in field-grown sorghum during heat waves (Pan *et al.*, 2022). This increase in *g*_sw_, coupled with decreased *A*_n_, led to a dramatic decrease of the intrinsic WUE (iWUE) in *Sen* at both growth temperatures. However, as *Tol* was able to maintain *A*_n_, its reduction in iWUE was less severe. *Sorghum bicolor* is known for its high drought tolerance and is often grown in countries where water is limited (Leff *et al.*, 2004). The smaller heat shock-induced reduction in WUE in *Tol* is a desirable trait that will become increasingly important if heat waves become more frequent in the near future (Weber *et al.*, 2018).

Both genotypes also down-regulated the respiratory machinery during heat shock, when measured at a standard common night temperature of 25 °C (Fig. 1G). Thermal acclimation via dark respiration is commonly observed across different plant species (Atkin and Tjoelker, 2003; Slot and Kitajima, 2015), and can be traced at the biochemical level, as discussed below. Down-regulation of dark respiration when combined with sustained photosynthesis, as seen in *Tol*, can provide the necessary carbon skeletons to sustain plant growth under heat shock (Wahid *et al.*, 2007; Prasad *et al.*, 2019).

### Elevated transcripts and metabolites related to heat tolerance prime Tol for heat shock

The two genotypes showed significantly different transcript and metabolite profiles both before and during heat shock, providing an array of avenues to investigate heat tolerance in sorghum. Utilizing knowledge from well-known model species (Wahid *et al.*, 2007), we are able to identify and explore known genes and metabolites related to heat tolerance. Two gene families that are known to underpin aspects of heat tolerance are HSFs and HSPs (Kotak *et al.*, 2007). HSFs largely regulate the induction of HSPs (Andrási *et al.*, 2021), and together their abundance can provide an insight into a plant’s short- and long-term response to heat stress. HSPs function as molecular chaperones to prevent protein denaturation and aggregation, as membrane stabilizers and antioxidants, and can also assist in protein refolding under stress conditions (Heckathorn *et al.*, 2002; Wang *et al.*, 2004; Allakhverdiev *et al.*, 2008). Here, the transcript abundance of HSPs drastically increased in response to heat shock in both genotypes. However, expression of several HSP transcripts was genotype specific, and *Tol* had significantly greater transcript abundance of numerous HSPs both before and during heat shock. Members of the HSP16.9 family had significantly elevated transcript expression in response to heat shock in *Tol*, but were expressed below 1 TPM during heat shock for *Sen*. Previous work has shown that HSP16.9 may be key to heat tolerance, where a single nucleotide polymorphism in the gene was able to differentiate heat tolerant and heat susceptible varieties of wheat (Garg *et al.*, 2012). We also found elevated transcripts in response to heat shock in *Tol* for two small HSPs (HSP26.8 and HSP15.1), the first of which is associated with a heat-tolerant variant of *Agrostis stolonifera* grass (Wang and Luthe, 2003). Small HSPs are often associated with thylakoids and the protection of the oxygen-evolving machinery of PSII against heat and oxidative stresses (Allakhverdiev *et al.*, 2008), and many of them have previously been shown to be up-regulated in response to heat in leaves of *Sorghum bicolor* variety BTx623 (Nagaraju *et al.*, 2020). Although the differences in response to heat shock were vast, another striking difference was seen when comparing the transcript expression of HSPs prior to the start of heat shock. An array of HSPs had significantly higher expression in *Tol*, when compared with *Sen*, before heat shock, including members of the HSP17 family and the highly conserved HSP70 family. Members of the HSP70 family are known to have a critical function in protecting cells from the detrimental effects of heat stress and can confer thermo-protective activity (Rousch *et al.*, 2004; Cho and Choi, 2009). Overexpression of members of the HS70 family has also been shown to confer heightened tolerance to heat stress (Wang *et al.*, 2016). The heightened transcript abundance of these HSPs may prime *Tol* to be able to respond to heat shock at a faster rate, helping alleviate the initial impact. Although HSP protein abundance cannot be inferred solely from the transcript abundance, such large differences would likely have some influence on their functioning.

There was some regulation of HSF transcripts in response to heat shock for both genotypes, but this was to a lesser extent than seen for HSP transcript abundance. This is likely due to sampling being carried out 6 d after the start of heat shock, meaning the initial signalling that HSFs are known to facilitate may no longer be as apparent (Guihur *et al.*, 2022). However, several HSFs still showed significant differences both in response to heat shock and between genotypes. Two of these, HSFA2 and HSFA6, have previously been shown to be key regulators of heat stress in wheat (Xue *et al.*, 2014). Members of HSFA2 and HSFA6 were significantly up-regulated in response to heat shock in *Sen* only, indicating this genotype is still responding to the heat shock several days into the treatment. In addition, the same HSFs were up-regulated in *Tol*, when compared with *Sen*, prior to the start of heat shock. Again, this would indicate *Tol* is primed to respond to heat shock, as the mechanisms by which it responds are functioning at an elevated baseline when compared with *Sen*.

Metabolites play a key role in maintaining homeostasis during abiotic stress, and constitutive elevated abundance of a suite of metabolites has been shown to be an effective way of conferring heat tolerance in plants (Serrano *et al.*, 2019). In the two genotypes assessed here, there were general increases in the main metabolite pools that enhance the synthesis of polyols, amino acids, amides, and quaternary ammonium derivatives in response to heat shock (Fig. 2). These increases largely represent groups that can function as osmolytes (e.g. amino acids and derivatives, polyols), but also many have been correlated with enhanced oxidative stress tolerance through the scavenging of free radicals and protection of enzymes against oxidative damage (Foyer and Shigeoka, 2011; Knaupp *et al.*, 2011). As such, these compounds can facilitate aspects of heat tolerance. As the function of soluble osmolytes in osmoprotection is to a large degree concentration dependent, we can venture an a priori higher osmotic regulation from these compounds (Yancey, 2005; Slama *et al.*, 2015). Osmoprotection is important because leaf relative water content usually decreases under heat shock (Al-Salman *et al.*, 2023). It has been suggested that polyols can decrease osmotic potential due to water-like hydroxyl groups, and so act as important osmoprotectants (Williamson *et al.*, 2002). Here, the concentration of the polyol galactinol was significantly increased in response to heat shock in both genotypes, but this response was to a greater extent in *Tol*. There was also enhanced concentration of two sugar osmolytes with strong osmoprotectant properties in both genotypes in response to heat shock: the disaccharide gentiobiose and the trisaccharide raffinose (Panikulangara *et al.*, 2004; Nishizawa *et al.*, 2008; Keunen *et al.*, 2013). However, this response was significantly heightened in *Sen*.

A decrease in the concentration of organic acids, particularly those involved in the TCA cycle, was identified in both genotypes in response to heat shock. However, this decrease was twinned with an up-regulation of the GABA shunt from the TCA cycle in both genotypes, with *Tol* increasing both *cis*-aconitate and 2-oxoglutarate 4-fold and glutamate (the most abundant amino acid) concentration 2-fold (Fig. 3). Glutamate provides the C and N for the biosynthesis of most other amino acids and GABA (Forde and Lea, 2007), and it is considered a signalling molecule that triggers heat thermotolerance (Toyota *et al.*, 2018; Li *et al.*, 2019). In fact, the concentration of many amino acids was significantly enhanced under heat shock only in *Tol*, including the first, second, and third most abundant amino acids (Glu, Ala, and Asn, respectively) and Ser. The accumulation of the non-protein amino acid GABA has been associated with carbon–nitrogen balance, ROS scavenging, and improving heat stress tolerance (Bouche and Fromm, 2004; Ansari *et al.*, 2021). Although stress may induce amino acid accumulation as a consequence of protein breakdown, the increased concentrations in *Tol* of the most abundant amino acids may have a beneficial effect during thermal acclimation as they can be used as compatible osmolytes or precursors for secondary metabolites (Krasensky and Jonak, 2012; Savchenko and Tikhonov, 2021).

Of the remaining metabolites measured here, several that are known to promote stress tolerance had significantly higher concentrations in *Tol* either prior to or during heat shock (when compared with *Sen*), including trimethylamine-*N*-oxide and *myo*-inositol (Mattoo *et al.*, 2015; Zhai *et al.*, 2016; Catalá *et al.*, 2021). Trimethylamine-*N*-oxide has been shown to act as a chaperone in plant cells, functioning as a protein-stabilizing osmolyte to maintain appropriate protein folding and enhancing plant performance during abiotic stress (Catalá *et al.*, 2021). *myo*-Inositol plays a dual function in stress- and non-stress-related pathways, providing membrane phospholipid derivatives and playing an important role in crosstalk between lipid and sugar signalling, as a regulator of SnRK1, and as a contributor to oxidative stress homeostasis (Valluru and Van den Ende, 2011). *Tol* presented a higher *myo*-inositol concentration during heat shock, compared with *Sen*. RFOs are additional osmolytes that can improve abiotic stress resistance (ElSayed *et al.*, 2014), several components of which (raffinose and galactinol) were up-regulated to a significantly greater extent in *Tol*, compared with *Sen*, in response to heat shock. Galactinol is the galactose donor in RFO synthesis and itself can confer membrane protection and radical scavenging during environmental stress (Krasensky and Jonak, 2012). Similar increases in RFO metabolites have been found in the C_4_ model *Setaria viridis* in response to increased growing temperatures (Zhang *et al.*, 2023, Preprint). RFOs are regarded as important molecules in stress response in plants for membrane stabiliziation, PSII protection, antioxidant and carbon storage, and signal transduction (Panikulangara *et al.*, 2004; Nishizawa *et al.*, 2008; Knaupp *et al.*, 2011; Sengupta and Majumder, 2015).

Heat shock can cause an increase in the fluidity of membranes and lipid peroxidation, which can lead to disintegration of the lipid bilayer and the down-regulation of membrane-bound protein activities (Upchurch, 2008; Sharma *et al.*, 2023). Plants increase the stability of membranes under heat stress through remodelling of the membrane lipid, which involves changes in membrane lipid composition and increases in the saturation level of membrane glycerolipids (Narayanan *et al.*, 2016; Higashi *et al.*, 2018). In our study, heat shock promoted a significant increase in the concentration of several building metabolites for membrane lipids, including glycerol-3-P (in both genotypes) and ethanolamine (2-aminoethanol; the second most abundant head group for phospholipids), which was significantly enhanced in *Tol* only. These changes suggest active remodelling of membrane lipids to maintain the membrane integrity of chloroplasts under heat shock in *Tol*. It is worth mentioning that changes in membrane properties lead ultimately to the expression of HSPs, which in turn confer additional heat tolerance (Horváth *et al.*, 2012).

### Regulation of metabolic processes allows plant functioning to continue during heat shock

Maintaining protein and membrane structure during heat stress not only requires protective mechanisms, but also efficient repair and replacement of proteins that become damaged (Wang *et al.*, 2018). However, as photosynthetic functioning is often inhibited (to some degree) during heat stress, balancing the availability of nutrients and their use can be delicate. Here, changes in the transcript profile of *Tol* suggest an up-regulation of several metabolic processes, changes not seen in *Sen*. Although significant reductions in the content of key sugars were consistent for both *Tol* and *Sen* during heat shock, as photosynthetic rate increased in response to heat shock in *Tol* it is possible increased metabolic processes could be maintained. Indeed, *Tol* was able to grow during the week of applied heat shock, presumably during the nights, a response not observed for *Sen* (Supplementary Fig. S2). Mechanisms that regulate metabolism under starvation or stress conditions are well studied, as they are integral to a plant’s ability to efficiently regulate its metabolic processes (Lemoine *et al.*, 2013). The protein complex that is known to be the central regulator for starvation is the sucrose non-fermenting-1-related protein kinase 1 (SnRK1) kinase complex (Wurzinger *et al.*, 2018). SnRK1 is known to respond to sugar starvation associated with darkness, nutrient deprivation, and stress, contributing to metabolic homeostasis and hence cell survival, providing a long-term framework for adaptation, growth, and development (Nunes-Nesi *et al.*, 2010). Here, few transcriptional changes were seen for the SnRK1 complex itself, either in response to heat shock or between genotypes. However, several transcript families and metabolites are known to allosterically regulate the SnRK1 complex (Hulsmans *et al.*, 2016). Several transcripts from the FCS-like zinc finger (FLZ) family have been shown to suppress the SnRK1 complex (Jamsheer *et al.*, 2018; Bortlik *et al.*, 2024). Here, three FLZ genes had significantly increased transcript abundance in response to heat shock in *Tol* only. Interestingly, these transcripts corresponded to the three FLZ genes previously linked to SnRK1 suppression (*FLZ3*, *FLZ6*, and *FLZ10*) (Jamsheer *et al.*, 2018; Bortlik *et al.*, 2024).

As SnRK1 responds to sugar starvation, it is also activated or supressed based on sugar status (Valluru and Van den Ende, 2011). The metabolite that is best known for its role in inhibiting the SnRK1 complex is trehalose-6-phosphate (Zhang *et al.*, 2009; Nunes *et al.*, 2013). Unfortunately, we were unable to measure trehalose-6-phosphate concentrations within this study. However, G6P is the trehalose-6-phosphate precursor, and elevated concentrations of G6P have also been shown to inhibit SnRK1 (Toroser *et al.*, 2000). A significant increase in G6P concentration in response to heat shock was apparent only for *Tol* (Fig. 4). Elevated G6P likely stimulates the production of *myo*-inositol as a precursor for phosphatidylinositol (membrane lipid) and galactinol production, the latter of which can lead to RFO synthesis (Fig. 4). These results indicate some suppression of SnRK1 signalling may be occurring in *Tol*. We suggest this suppression is required to avoid inhibition of growth via the SnRK1 complex as the abundance of key sugar compounds decreases during heat shock. The length of time this response could persist would require further investigation. However, as a short-term response to heat shock, it appears to allow the plant to avoid inhibition by utilizing carbohydrate reserves and/or newly synthesized sugars through photosynthesis. Furthermore, *Tol* plants exposed to the heat shock produced an apparently healthy inflorescence (Supplementary Fig. S2). These results strengthen the idea that *Tol* is primed through osmoprotectants, ROS scavengers, chaperones, and complex signalling pathways linked with plant growth regulators that enable tolerance to the imposed heat shock and provide a metabolite profile that can be screened against to identify *S. bicolor* genotypes that are naturally primed to respond to heat stress (Fig. 8).

### Conclusion

Understanding how plants acclimate to heat shock is becoming increasingly important, considering the increased frequency and intensity of heat shocks in current climates. As sorghum is regularly grown in hot and arid areas of the world, elevated temperatures may cause detrimental losses in crop yield. Here, we investigated the acclimatory strategies of two sorghum genotypes, one sensitive (*Sen*) and one tolerant (*Tol*) to heat shock, by exposing them to a 6 d heat shock, with the last 3 d reaching 45 °C. The underpinning response of *Tol* exhibited several intriguing constitutive and inducible components. Prior to and during heat shock, an increased abundance of HSP transcripts was noted for *Tol*, when compared with *Sen*. During heat shock, evidence of maintained metabolic processes and up-regulated photosynthetic processes were identified for *Tol*, even as the abundance of several hexose sugars became depleted. In addition, several key metabolites were up-regulated in *Tol* in response to heat shock, some of which are recognized osmoprotectants and ROS scavengers and others are involved in membrane lipid stability. We suggest this suite of responses play a role in maintaining plant function during heat shock in *S. bicolor* to replace and repair damaged proteins, many of which are associated with cell membranes. We also suggest this is facilitated by the suppression of the SnRK1 complex via increased abundance of both transcripts (FLZ family) and metabolites (G6P) known to supress the complex. Together, our results highlight pathways, genes, and metabolites that can be harnessed to select *S. bicolor* genotypes (and potentially genotypes within other species) with more favourable traits in future breeding trials.

## Supplementary data

The following supplementary data are available at *JXB* online.

Fig. S1. Glasshouse temperature data taken before and during heat shock for low temperature (LT) treatment.

Fig. S2. Pictures of each genotype and evidence of continued growth in *Tol* during heat shock.

Fig. S3. Normalized NanoString count percentage change of known heat shock proteins.

Dataset S1. Transcript per million (TPM) values for each *Sorghum bicolor* gene identified within this experiment.

Dataset S2. Significantly differentially expressed (DE) transcripts and gene ontology (GO) over-representation in the identified DE transcripts.

Dataset S3. *Sorghum bicolor* accessions for each gene of interest.

Dataset S4. List of nCounter and Capture probes used.

Dataset S5. Metabolites measured by GC-MS and CE-MS both before and during heat shock for both genotypes grown at LT (22 °C).

erae506_suppl_Supplementary_Figures_S1-S3

erae506_suppl_Supplementary_Datasets_S1

erae506_suppl_Supplementary_Datasets_S2

erae506_suppl_Supplementary_Datasets_S3

erae506_suppl_Supplementary_Datasets_S4

erae506_suppl_Supplementary_Datasets_S5

## Data Availability

Raw RNA sequencing reads are available within GenBank under the BioProject accession PRJNA1074961.

## Abbreviations:

B: before heat shock
D: during heat shock
DE: differentially expressed
G6P: glucose-6-phosphate
HSF: heat shock factor
HSP: heat shock protein
HT: high temperature
LT: low temperature
PCA: principal component analysis
RFO: raffinose family oligosaccharide
TPM: transcripts per million
WUE: water use efficiency
