# Supplementary material for: Multi-omic profiles of Sorghum genotypes with contrasting heat tolerance connect pathways related to thermotolerance
Source: J Exp Bot. 2024 Dec 19;76(17):4980–98. doi: 10.1093/jxb/erae506 (PMC12587425; doi:10.1093/jxb/erae506)
Supplement: erae506_suppl_Supplementary_Figures_S1-S3 [file erae506_suppl_supplementary_figures_s1-s3.pdf]

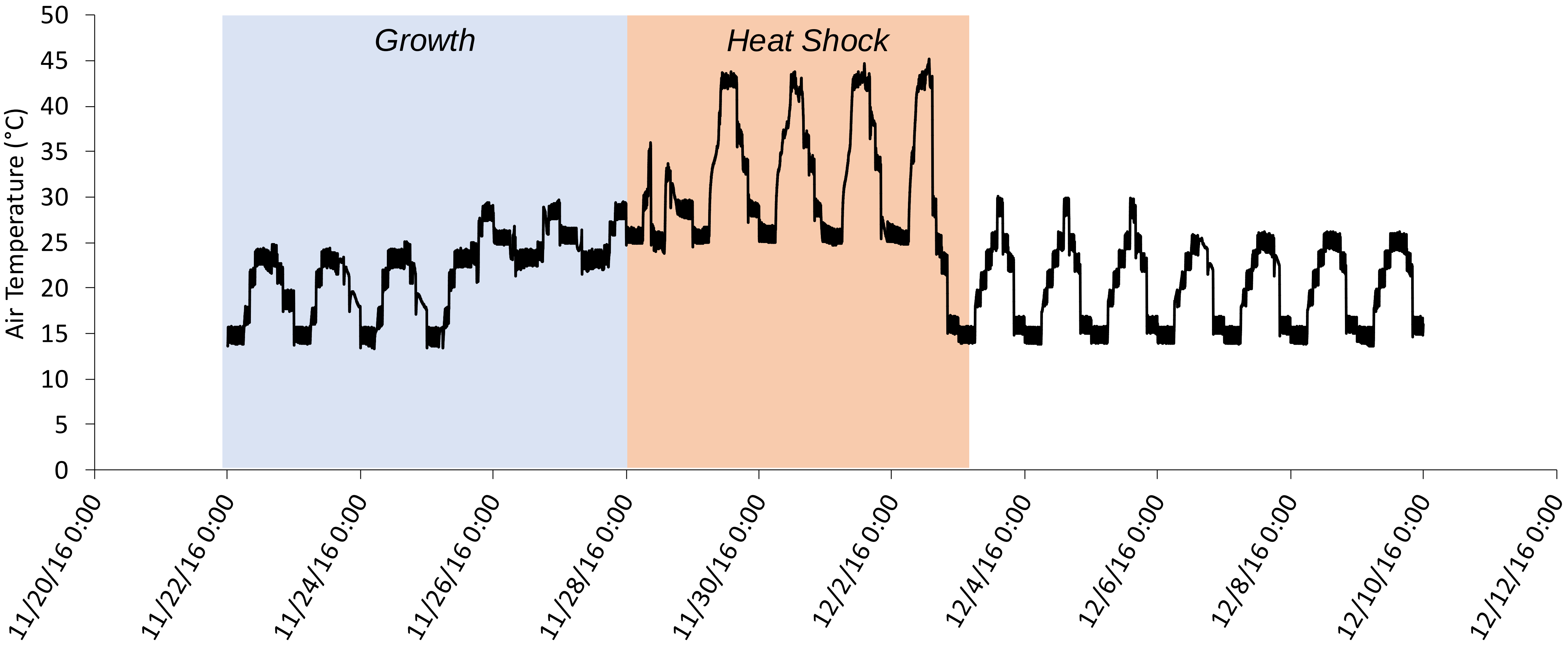


Supplementary Figure S1 – Glasshouse temperature data taken before and during heat shock for low temperature (LT) treatment.

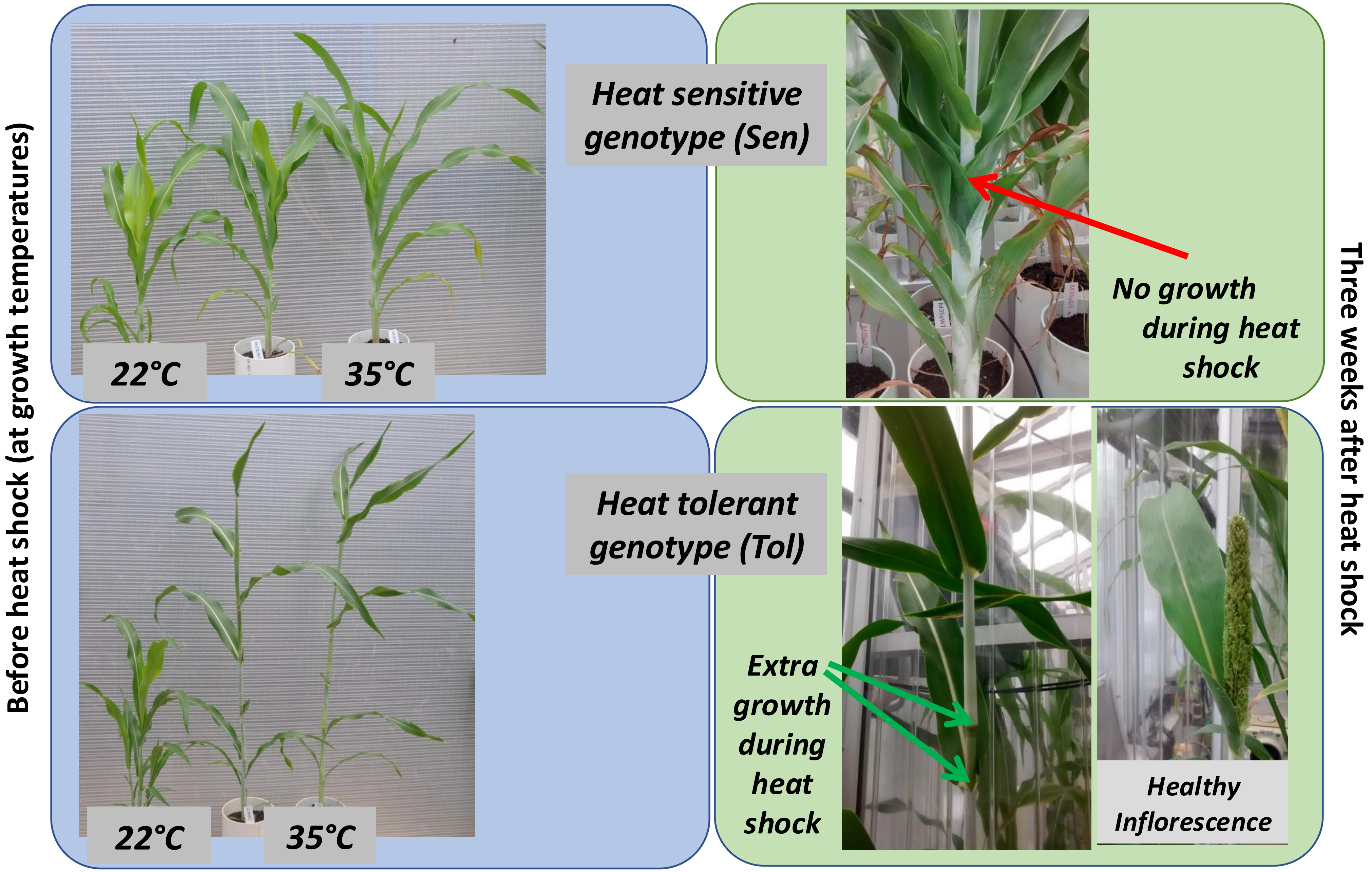


Supplementary Figure S2 – Pictures of each genotype and evidence of continued growth in *Tol* during heat shock.

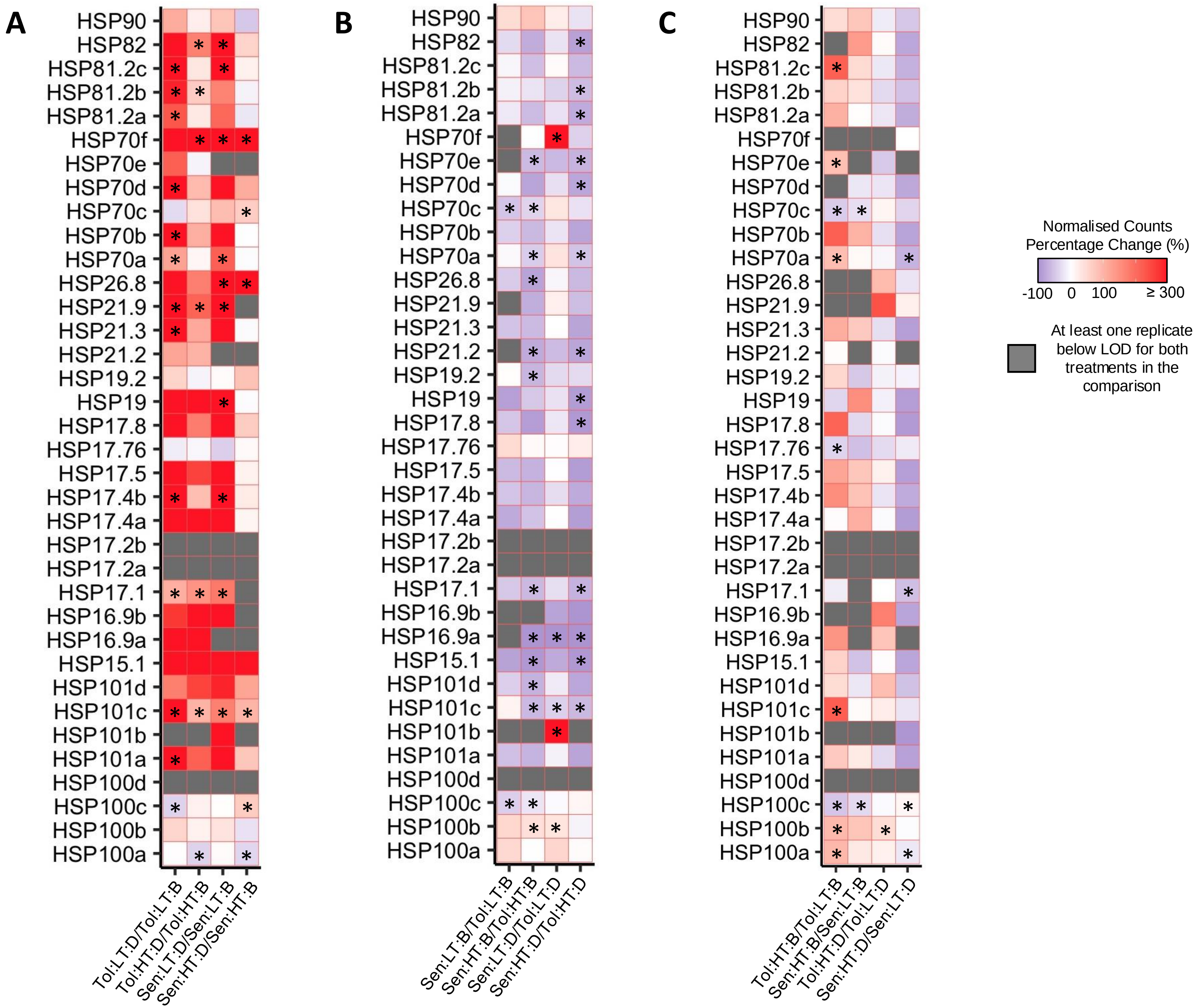


Supplementary Figure S3 – Normalised NanoString count percentage change of known heat shock proteins. Comparisons shown are (**A**) in response to heat shock, (**B**) between genotypes and (**C**) between growth temperatures. Group comparisons shown as B/A, with percentage change calculated via the calculation ((B-A)/A) x 100. Sen – sensitive genotype, Tol – tolerant genotype, LT – low temperature (22°C), HT – high temperature (35°C), B – before heat shock, D – during heat shock, LOD – level of detection. *S. bicolor* accession numbers for each gene can be found in Supplementary Dataset S3.
